# Lessons learned in the implementation of a study to evaluate intestinal colonization with antibiotic-resistant bacteria in communities and hospitals in India

**DOI:** 10.3389/fpubh.2026.1851582

**Published:** 2026-08-26

**Authors:** Girish Kumar Chethrapilly Purushothaman, Sathya Narayanan Gopalakrishnan, Tarun Bhatnagar, Swathi Subbiah Sankarraj, V. Sindhuja, Valan A. Siromany, Daniel VanderEnde, Ahmed Babiker, Paul Malpiedi, Susan Bollinger, Ashley Styczynski, Rachel M. Smith

**Affiliations:** 1 Division of Laboratory Sciences, Indian Council of Medical Research National Institute of Epidemiology, Chennai, India; 2 Faculty of Medical Research, Academy of Scientific & Innovative Research, Ghaziabad, India; 3 ICMR School of Public Health, Indian Council of Medical Research National Institute of Epidemiology, Chennai, India; 4 Division of Healthcare Quality Promotion, Centers for Disease Control and Prevention, Atlanta, GA, United States; 5 Division of Infectious Diseases, Department of Internal Medicine, Rush University, Chicago, IL, United States

**Keywords:** antibiotic-resistant bacteria, ARCH, challenges, colonization, implementation, India, lessons learned

## Abstract

**Introduction:**

Measuring colonization with antibiotic-resistant bacteria (ARB) and associated risk factors in community members and hospitalized patients is crucial to combating the spread of ARB. The Antibiotic Resistance in Communities and Hospitals (ARCH) study, conducted in multiple countries, aimed to assess the prevalence of and risk factors for intestinal colonization with ARB of public health importance.

**Methods:**

The prevalence and risk factors of intestinal colonization with ARB was determined through and participant surveys among 757 community and 556 hospitalized adult individuals and bacterial isolation from their stool specimens. This paper focuses on the implementation experience of the ARCH study in India.

**Results:**

Implementing robust AMR studies in low- and middle-income country settings is very challenging. Socio-cultural factors likely resulted in a high refusal rate (Community—49%; Hospital—59%) for stool sample collection. In the laboratory, discordant results from Vitek®2 Compact had to be confirmed using reference methods. Study implementation was further complicated by the COVID-19 pandemic, leading to delays in participant recruitment, suspension of fieldwork during lockdowns, and interruption of laboratory testing due to supply chain limitations. Taboos around stool collection and handling were overcome with the help of members of the local administrative body in the community and medical staff in the hospitals. Conducting an initial pilot exercise followed by periodic reviews of the study’s progress proved beneficial in limiting errors and challenges during the study, and precautionary steps were taken during data management to ensure data quality.

**Conclusion:**

Implementation challenges were addressed through early stakeholder engagement, pilot testing of study procedures, robust laboratory quality assurance mechanisms, and integrated electronic data management systems. Lessons learned from this study may inform future AMR surveillance initiatives in resource-limited settings.

## 1 Introduction

Infections with antibiotic-resistant bacteria (ARB) can result in severe disease, prolonged hospitalization, increased duration of treatment, excess healthcare costs, and death (1, 2). ARB are increasingly identified in community settings, suggesting ARB reservoirs may exist outside healthcare facilities (1–4). Colonization with ARB can predispose a person to antibiotic-resistant infections and be a source for onward transmission. Thus, understanding the prevalence of and risk factors associated with intestinal colonization with ARB represents an opportunity to identify interventions that may mitigate the impact of ARB across healthcare and community settings. Current estimates of AMR in low- and middle-income countries (LMICs) are largely based on limited data from incident clinical infections and are impacted by variability in utilization of healthcare and diagnostic services. Colonization studies complement clinical surveillance data by providing a more comprehensive assessment of the community reservoir of ARB, generating insights into the potential burden of AMR-associated disease and informing prevention and control strategies in both community and healthcare settings (5).

The Antibiotic Resistance in Communities and Hospitals (ARCH) research consortium, which includes sites in 6 countries across 4 continents, uses cross-sectional surveys to estimate population-based prevalence of ARB colonization and evaluate risk factors associated with colonization (6). The consortium used a common core protocol across all participating countries, with limited site-specific adaptations (6). In India, we collected relevant demographic and epidemiologic data through participant surveys and evaluated intestinal colonization with extended-spectrum cephalosporin-resistant Enterobacterales (ESCrE), carbapenem-resistant Enterobacterales (CRE), and colistin-resistant Enterobacterales (Col-RE) using bacterial isolation from stool specimens (7).

Conducting studies of antimicrobial resistance (AMR) burden in LMICs is essential to fill gaps in global estimates and inform prevention efforts. However, there are unique aspects of the LMIC context that pose additional challenges in collecting this type of data. There are limitations in the laboratory and diagnostic infrastructure and the availability of skilled human resources. As a populous, geographically, and culturally diverse country, India has complex healthcare delivery practices and socio-cultural factors that can create difficulties in implementing studies that require biological specimens and/or personal information. In India, AMR data are primarily from large hospital-based surveillance networks, while relatively few studies have examined colonization with ARB, particularly in community settings. Further, there is limited published literature describing the operational, laboratory, logistical, and socio-cultural challenges associated with implementing such studies in LMICs. The present paper addresses the existing gap in the literature by presenting the challenges encountered and lessons learned during the implementation of the ARCH study in India, with the aim of informing future AMR colonization studies in similar settings.

## 2 Materials and methods

We conducted community and hospital surveys in a semi-urban region of Chennai between November 2020 and March 2022. Eligible participants were adults ≥18 years from the community and hospitalized patients meeting predefined inclusion criteria. Assuming a 20% prevalence of colonization with at least one ARB phenotype, the target sample sizes were 750 community participants and 509 hospitalized patients. We calculated frequencies and proportions for participant enrollment, stool specimen collection, bacterial species isolated from the collected stool samples and the target resistant phenotypes (ESCrE, CRE, Col-RE). We have described the eligibility criteria, sampling design, sample size and statistical analysis in detail elsewhere (7).

### 2.1 Implementation of the antibiotic resistance in communities and hospitals study protocol in India

#### 2.1.1 Regulatory and administrative approvals

The United States Centers for Disease Control and Prevention (CDC) partnered with the Indian Council of Medical Research (ICMR) - National Institute of Epidemiology (NIE) in Chennai, a public health research institute under ICMR to implement the ARCH cross-sectional colonization study protocol modified to the Indian context (Table 1). The protocol underwent technical and ethical reviews by the ICMR-NIE Scientific Advisory Committee, the Institutional Human Ethics Committee, and the Health Ministry’s Screening Committee of the Government of India. Implementation of the project required a multi-disciplinary team of microbiologists, epidemiologists, and data experts. This was achieved through a strong collaboration between researchers from ICMR-NIE and U. S. CDC as well as a dedicated team of field assistants hired for the project.

**Table 1:** Antibiotic Resistance in Communities and Hospitals (ARCH) protocol and its modifications in India.

Particulars	ARCH protocol	Modifications to ARCH in India	Rationale
ARB^*^ of interest	Extended-spectrum cephalosporin-resistant Enterobacterales (ESCrE) Carbapenem-resistant Enterobacterales (CRE) Methicillin-resistant *Staphylococcus aureus* (MRSA)	ESCrE and CRE (MRSA Excluded)	Enterobacterales were prioritized due to their key role in driving antibiotic resistance spread and their high prevalence among clinically significant pathogens monitored across national AMR surveillance systems in India
Optional ARB	Colistin-resistant Enterobacterales (Col-RE) Carbapenem resistant *Pseudomonas aeruginosa* (CRPA)	Col-RE	
Sampling method for community participants	Simple random sampling or cluster sampling	Simple random sampling	Availability of previously enumerated population
Sampling method for hospital participants	Stratified with proportional allocation	Available patients were enrolled till the sample size was achieved	COVID-19 restrictions limited the number of hospital wards accessible for participant enrolment
Sample type	Stool or rectal swab + nasal swab	Stool samples	Study focused on gut colonization with Enterobacterales. Stool samples were collected due to better social acceptance

*ARB, Antibiotic resistant bacteria.

#### 2.1.2 Field activities

The project implementation team including field investigators underwent training to use Open Data Kit (ODK Collect app, v2021.2.4), which is an open-source Android application that was installed on tablets. After the training, they conducted mock interviews to practice administering the study questionnaire. Following this, the field staff carried out pilot tests involving 15 individuals in community and hospital settings to ensure comprehension of the questions, determine the most effective procedures and tools for participant recruitment, and identify challenges in stool sample collection. The stool specimens then underwent testing at the NIE microbiology laboratory. This pilot exercise enabled the project implementation team to gain hands on experience in approaching participants; administering the study questionnaire using Open Data kit application; collecting, transporting, and testing laboratory specimens as per the study protocol (7).

##### 2.1.2.1 Community component

The community survey was conducted in a semi-urban area of Chennai. Prior to the commencement of the study, field workers met with local administrative body (Village Panchayat) members. This built trust between field investigators and the study population and sensitized them to stool sample collection efforts. Households were selected through simple random sampling from a pre-enumerated Demographic and Health Surveillance (DHS) cohort. From each selected household, one eligible adult (≥18 years) was randomly chosen for enrolment. If the selected participant was unavailable, the survey team arranged a telephone interview to complete the questionnaire.

##### 2.1.2.2 Hospital component

Two hospitals (one government and one private) were selected based on locality, healthcare service linkage to the community, bed capacity, and occupancy rate. Throughout the study period (enrollment, data, and sample collection), one team, along with a sample transporter, was available at each hospital. Study nurses sensitized patients and facilitated participant enrollment and stool collection. We conducted daily surveillance and all patients ≥18 years of age admitted to hospital wards (excluding COVID-19 wards) were approached for enrollment. A questionnaire was administered to inpatient study participants.

#### 2.1.3 Laboratory procedures

Participants received a stool collection kit comprised of tissue paper, a stool hat (Thermo Fisher Scientific, USA), disposable gloves, a leak-proof transport container with spatula/scoop (HiMedia, India), zip-lock bags, and a cool box (thermocol box with frozen gel packs). These kits were collected by sample transporters from community and hospital participants the following morning and transported to the NIE laboratory within 1 h for processing. Field, laboratory, and data teams used a mobile phone-based encrypted instant messaging application to communicate and coordinate study activities. Training for laboratory staff on the preparation of selective chromogenic media (i.e., ESBL, mSuperCARBA, and COL-APSE CHROMagars; CHROMagar™, France) was conducted at a tertiary care government teaching hospital where this was routinely performed.

Stool specimens were inoculated on the CHROMagar plates prepared from dehydrated powders following the manufacturer’s instructions. Following overnight incubation, up to 3 unique colony morphotypes suggestive of Enterobacterales species were selected from each plate (8–10). Isolate identification and antimicrobial susceptibility testing (AST) were performed on the Vitek (bioMérieux, France) platform using the GN-ID and N281 AST cards, respectively. The AST result interpretation was determined using Clinical and Laboratory Standards Institute (CLSI) 2022 breakpoints (11). Quality control (QC) of the CHROMagar and Vitek testing was performed as per manufacturer instructions using American Type Culture Collection (ATCC) strains (8–11).

#### 2.1.4 Data management

Field study data were transferred weekly from tablets to a static server. Data validation for missing values, out-of-range entries and other data inconsistencies was performed using Microsoft Excel. Errors were communicated to the field team for verification and, if needed, correction. The bacterial ID and AST results were exported from the Vitek 2 Compact within a 30-day post-testing period (default setting). Exported data were reviewed within 3 days. The field and laboratory data were merged prior to data analyses. Data security was ensured through password-protected tablets and computers and use of a secure server. A digital archive of the database was backed up in an encrypted external hard drive.

## 3 Results and discussion

**Figure 1:**
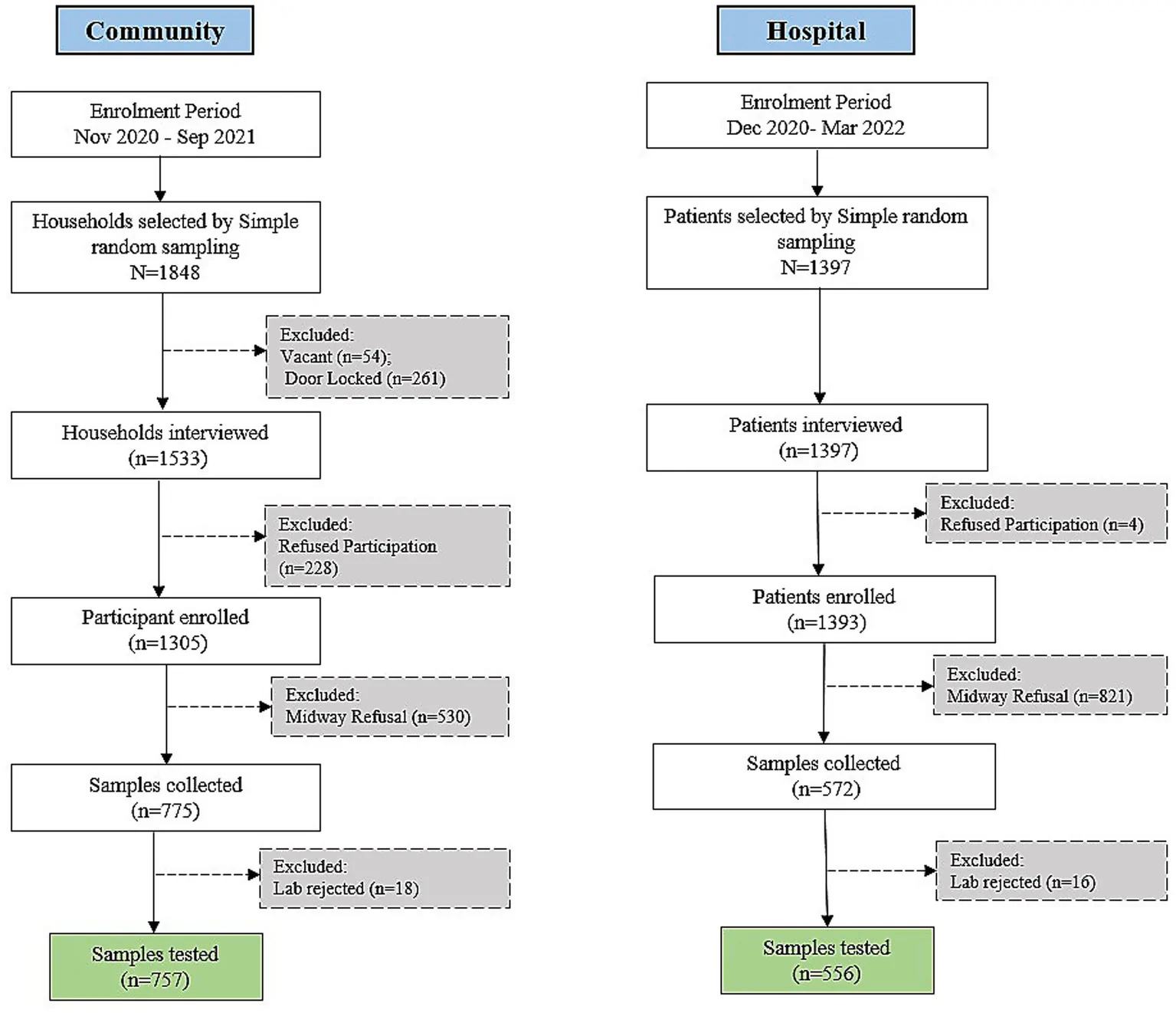
Flow chart summarizing participant enrolment from the community and hospital in a semi-urban setting in South India: 2020–2022.

Between November 2020 and March 2022, 1,305 community participants and 1,393 hospitalized patients were enrolled. Stool specimens were collected from 757 (58%) community participants and 556 (40%) hospitalized patients. At least 1 target resistant phenotype (ESCrE/CRE/Col-RE) was detected in 77.7% of participants from the community and 90.3% of hospitalized participants. The enrolment summary, and study timeline are summarized in (Figure 1).

Challenges, observations, best practices, and opportunities.

**Figure 2:**
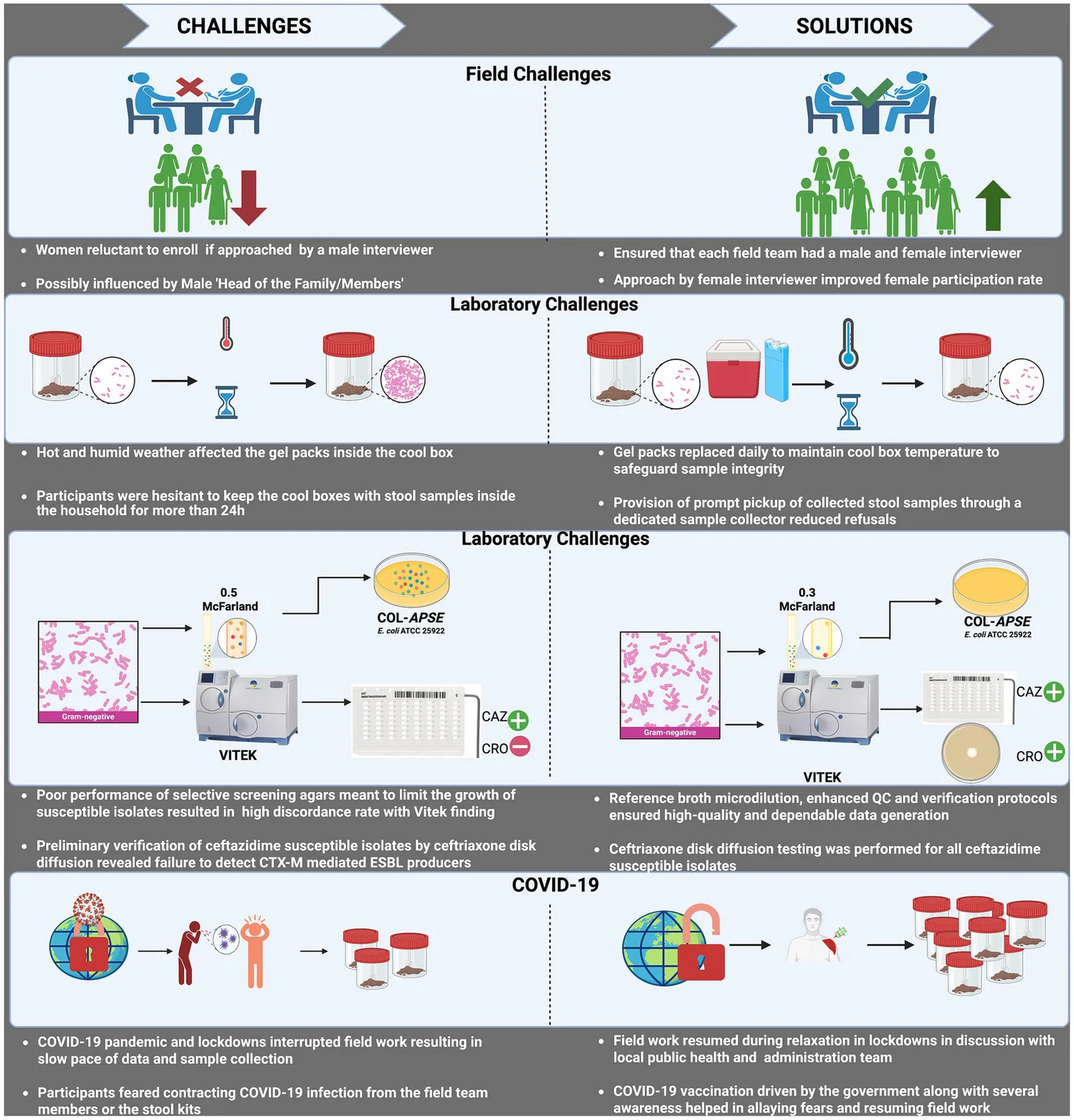
Challenges and lessons learned/solutions from the implementation of the Antibiotic Resistance in Community and Hospitals (ARCH) study in India. Created with BioRender.com.

Several challenges at various stages, including socio-cultural barriers, operational factors, laboratory challenges, and procurement difficulties, arose in the implementation of the project, necessitating real-time troubleshooting and protocol adaptations (Figure 2).

### 3.1 Field challenges

#### 3.1.1 Project approval

The process of obtaining scientific, ethical, and in-country approvals took around 18 months. Although rectal swab collection was part of the initial ARCH protocol, logistical complexities and concerns about social acceptability led the investigators to adopt a whole-stool collection approach. The revised protocol was implemented following receipt of all required approvals.

#### 3.1.2 Case finding and data collection

Of the 1848 randomly selected households, 1,533 households were available for enrolment in the study (Figure 1). Of the available households, 51% consented for interview and providing stool samples whereas the remaining 49% (758/1533) refused. Similarly, in the hospital among the 1,393 enrolled inpatients the refusal rate was 59% (825/1397) and 41% consented for interview and providing stool samples. High refusal rates delayed meeting participant enrolment targets by 3 months. Although reasons for refusal were not systematically collected, anecdotal observations by field staff suggested that participant refusal to provide stool samples may have been influenced by socio-cultural factors, including discomfort with stool collection and with storing stool containers within the household premises. The opinion of the father, husband, or older brother serving as the head of the household was viewed as irrevocable in the patriarchal nature of the study population. This influenced both enrollment and stool sample collection. Among the participants enrolled during the pilot, male field investigators found some heads of households and female participants refused to discuss stool collection, resulting in a refusal to participate. Subsequently, we assigned a male and female interviewer to each field team to interact with the participants of the corresponding sex. This improved the participation rate in the study.

#### 3.1.3 Operational factors

Delays were experienced in achieving the required hospital sample target when patients did not use the sample collection kit, were discharged prior to sample collection, were lost after transfer to a different ward, or could not give a sample due to constipation. Support from hospital staff enabled tracking of participants transferred to other wards within the study hospital and thereby improved the stool collection rate.

### 3.2 Laboratory challenges

#### 3.2.1 Sample collection

Sample leakage was identified as an early challenge during study implementation and was promptly addressed through reinforced field team training with emphasis on proper capping and safe transportation of stool containers. There were also delays in sample collection from community participants who could not provide the sample within 24 h. Chennai’s tropical climate, characterized by hot and humid weather, posed a challenge to maintaining sample integrity. During the pilot exercise, the team found that the cool box frozen gel packs did not maintain the desired temperature (2–8 °C) beyond 24 h. To address this, if a delay in stool collection was anticipated or a participant reported failure to collect a sample within 18-24 h of enrollment, the gel packs were replaced by a field team member. To improve quality control, a “cool box temperature check” was added to the stool specimen data collection form. Some participants hesitated to keep the cool box containing their stool samples within their premises for long durations. This was resolved by following up and arranging sample pick up when the participant confirmed its collection telephonically.

#### 3.2.2 Chromogenic agar performance and discordance with Vitek

Initial QC testing of the CHROMagar COL-APSE agar revealed that *E. coli* American Type Culture Collection 25,922, a colistin-susceptible quality control strain that was expected to be inhibited, grew on the culture plates. The technical team contacted the CHROMagar manufacturer who suggested using an inoculum adjusted to match 0.3 instead of the standard 0.5 McFarland opacity standard. This resolved the performance of the QC strain and testing resumed.

Ceftazidime is the only extended-spectrum cephalosporin included on the Vitek N281 AST card. A high level of discordance was observed between results from ESBL selective chromogenic agar and ceftazidime AST. Of the 1,269 isolates recovered from 1,010 participants on the ESBL agar, only 581 (45.8%) were resistant to ceftazidime on the Vitek. We hypothesized that this high discordance was due to the global predominance of CTX-M type ESBLs (12), which preferentially hydrolyze ceftriaxone and cefotaxime over ceftazidime (13). To test this hypothesis, a preliminary testing for a random sample of 10 ceftazidime susceptible isolates that grew on the CHROMagar ESBL was screened by ceftriaxone disk diffusion to ensure the identification of all ESCrE with CTX-M type ESBL (14). This revealed a high level of ceftriaxone resistance (80%, 8/10). This led us to perform the ceftriaxone Kirby-Bauer disk diffusion assay for all ceftazidime susceptible isolates (*n* = 1809), of which 51.5% were found to be ceftriaxone resistant. This experience emphasizes the importance of including ceftriaxone and/or cefotaxime in AST panels or testing for accurate identification of ESCrE.

In addition, a low level of concordance (25.3%) was observed between CHROMagar mSuperCARBA and Vitek results. This may reflect growth of susceptible organisms from primary specimen inoculation, potentially influenced by factors such as inoculum size, specimen matrix effects (including pH changes from stool specimens), and variability in media handling or incubation conditions. Overall, this experience underscores the value of conducting verification studies of the chromogenic screening agars during the pilot phase, as early identification of discordance could guide the selection of a more reliable screening method.

#### 3.2.3 Colistin confirmatory testing

Due to the physical properties of polymyxins (large cationic molecules), including colistin, and the possibility for major and very major errors, the CLSI guidelines and the European Committee on Antimicrobial Susceptibility Testing (EUCAST) do not recommend using conventional AST methods such as disk diffusion, gradient diffusion, or commercial automated AST platforms like Vitek. Studies have demonstrated that Vitek 2 colistin results are unreliable due to high false susceptibility (15). CLSI and European Committee on Antimicrobial Susceptibility Testing recommend performing broth microdilution (BMD) for colistin (10). BMD is labor-intensive and is not routinely performed in most clinical microbiology laboratories in India. We opted to perform colistin BMD on only a subset of isolates due to financial and time constraints. All colistin-resistant isolates (MIC ≥ 4 mg/L, *n* = 32) and a subset (about 10%) of the intermediate isolates (MIC ≤ 2 mg/L, *n* = 307) characterized using Vitek were retested using BMD to improve accuracy in AMR prevalence estimates. Since colistin BMD was not previously done at NIE, it was necessary to get the laboratory staff trained at an in-country laboratory where the assay was routinely performed. This was achieved with training support from the national AMR reference laboratory at the National Center for Disease Control, New Delhi. Following the BMD training, the ARCH laboratory team additionally completed a verification study using a panel of previously characterized Enterobacterales, as outlined by CLSI (16). Confirmatory testing by BMD established resistance in 9.4% of Vitek-established resistant isolates and 2.6% of the tested non-resistant isolates. This demonstrated the unreliable performance of Vitek for determining colistin resistance, emphasizing the importance of doing colistin confirmatory testing when relying on Vitek-based AST.

#### 3.2.4 Quality control

Laboratory testing quality was ensured through adherence to manufacturer-recommended QC procedures for CHROMagar, Vitek ID, and AST. Despite successful verification of Vitek GN ID cards for Enterobacterales, there was occasional misclassification (0.5%) of certain Gram-negative bacteria such as *Acinetobacter* spp., *aerotolerant Clostridium* spp., *and Shigella* spp., with low-level discrimination in the Vitek ID output. These misclassifications were resolved through confirmatory standard biochemical testing.

### 3.3 Procurement challenges

In our study, we could not obtain the ceftriaxone antibiotic disks validated in multiple countries due to import restrictions and complex licensing formalities. Therefore, disks sourced from a local manufacturer underwent CLSI-recommended verification procedures using *E. coli* ATCC 25922. The zone diameters obtained with the locally sourced disks consistently fell within the CLSI-prescribed QC ranges. Based on these verification results, the disks were considered acceptable for use in the study. Our above experience highlights the need for access to and use of strict and verification protocols to ensure high-quality and dependable data generation. A critical component to generating reliable laboratory data is access to quality-assured reagents and materials, such as those provided by International Organization for Standardization (ISO) certified manufacturers (17). In LMICs, the problem of obtaining diagnostic and medical supplies may be difficult due to supply chain issues such as non-availability of ready stock supplies at local distributors, shorter shelf life for perishable products/kits, and longer turnaround times in procurement policies (18, 19). To address these challenges, we prepared detailed supply lists and initiated procurements before implementation of the study. Despite this preparedness, challenges were faced because of the COVID-19 pandemic.

### 3.4 Data management challenges

We did not encounter any major challenges in data management. Data monitoring measures such as daily logs were maintained by the field and laboratory teams to monitor enrollment and laboratory testing. Real-time data monitoring enabled timely detection of discrepancies, followed by quick resolutions and preventive actions. Weekly meetings, conducted with the participation of all field and laboratory team members, were helpful in consolidating the data, updating the study progress, sharing best practices, and addressing challenges.

Understanding the need for high-quality data, validation checks were built into the Open Data Kit (ODK) application at the point of data entry to minimize transcription and entry errors. Firstly, most questions were menu-based checkboxes or dropdown interfaces. Second, because data exported from Vitek contained only MIC values of the antibiotics for the isolates, syntaxes were written in R Studio to interpret the MIC values. Thirdly, separate MS Excel spreadsheets were used for laboratory and risk factor data. This separation allowed for one-to-many merges required for the laboratory data since a single participant might have up to nine possible organisms selected from three chromogenic agar plates. The laboratory data were merged with the field data only after the final interpretations of the ARB isolates were available.

### 3.5 Challenges related to the COVID-19 pandemic

The study was scheduled to start in March 2020; however, study initiation was delayed due to the COVID-19 pandemic and containment measures such as strict lockdowns beginning in March 2020. Following the relaxation in containment measures after the first COVID-19 wave, fieldwork began in November 2020. The study continued until April 2021, when new lockdowns were announced. This time, the field team limited the number of household visits, used appropriate personal protective equipment (PPE), and performed strict hand hygiene practices during sample collection. This resulted in a slower pace of data and sample collection.

During the study, community participants were hesitant to engage with study staff due to fear of contracting COVID-19 from the staff and the stool kits. This hesitancy did not change despite efforts by the team to explain the safety measures being taken to prevent virus transmission. In the hospitals, the field teams had difficulty accessing the hospital wards, as most of them were converted into COVID-19 wards. Fieldwork slowed when field team members contracted COVID-19. Following the rollout of the COVID-19 vaccination, improved public awareness, relaxation of lockdowns, and improved accessibility to community and hospital participants enabled completion of field and hospital enrolment. The COVID-19 pandemic also led to disruptions in the supply chain that impacted CHROMagar procurement. This necessitated the use of previously purchased but expired CHROMagar during the supply chain disruptions. All the primary isolates tested using the expired CHROMagar were reassessed when fresh media supplies became available.

### 3.6 Unresolved issues

The enrollment of the participants was affected by the COVID-19 pandemic, which could have impacted prevalence estimates. Support to public health research during public health pandemics requires international coordination and lessons learned from COVID-19 should be applied to future pandemics. Additionally, restrictions on procurement of certain supplies hindered access to quality reagents and materials. As public health research depends on resilient supply chains, it is important that researchers from LMICs continue to raise awareness with their governments and particularly ministries of health for smoother procurement processes for internationally sourced research materials.

### 3.7 Limitations

We have previously reported a high refusal rate in our study (7), which could have introduced selection bias, as households or inpatients who agreed to participate might have systematically differed from those who declined. Given the resource and budget constraints of conducting the study in LMIC settings, where phenotypic methods remain the mainstay of AMR diagnosis and molecular testing capacity is often limited, molecular characterization of discordant isolates was not included in the study protocol. Consequently, we could not determine whether the discordant results reflected false-positive findings on chromogenic agar or false-negative findings by Vitek. Although, a subset of isolates was retested using BMD, Col-RE prevalence and co-colonization may be underestimated because of the reliance on Vitek results. Finally, the challenges and lessons learned in this study may not be generalizable across all LMIC settings.

## 4 Conclusion

ARB colonization studies in LMIC settings can be operationally challenging. Key challenges and lessons learned from this study spanned logistic, cultural, and operational aspects of study implementation and enrollment to the technical aspects of specimen collection, transport, and laboratory analysis. Careful preplanning, including community sensitization, appropriate team composition, piloting key aspects of the study and laboratory methods such as verification protocols of chromogenic agars, can help identify challenges early. Research studies and AMR surveillance activities conducted in LMICs should ensure that reference clinical microbiology procedures are available to confirm out-of-range or unexpected results. Additionally, flexibility and innovation to identify and address challenges, even in unforeseen situations like the COVID-19 pandemic, are critical to advance public health research. Collaboration between research institutions, as in the ARCH study in India, is an excellent way to leverage the strengths of different groups to contribute collective expertise in areas with limited resources. The best practices identified through the ARCH study could be scaled through integration into existing national surveillance networks, strengthening of laboratory and workforce capacity, adoption of standardized protocols, and robust data management systems. Such approaches would reduce dependence on specialized project-specific infrastructure while supporting sustainable AMR surveillance. The challenges identified and managed during this successful study implementation in India may help inform other epidemiologic studies in India or other LMICs with similar socio-cultural environments, health system characteristics, and resource restrictions.

## Data Availability

The data supporting the conclusions of this article may be made available by the corresponding author upon reasonable request.
